# Supplementary figures and images for: Xylem Anomalies as Indicators of Maladaptation to Climate in Forest Trees: Implications for Assisted Migration
Source: Front Plant Sci. 2020 Feb 27;11:208. doi: 10.3389/fpls.2020.00208 (PMC7057245; doi:10.3389/fpls.2020.00208)

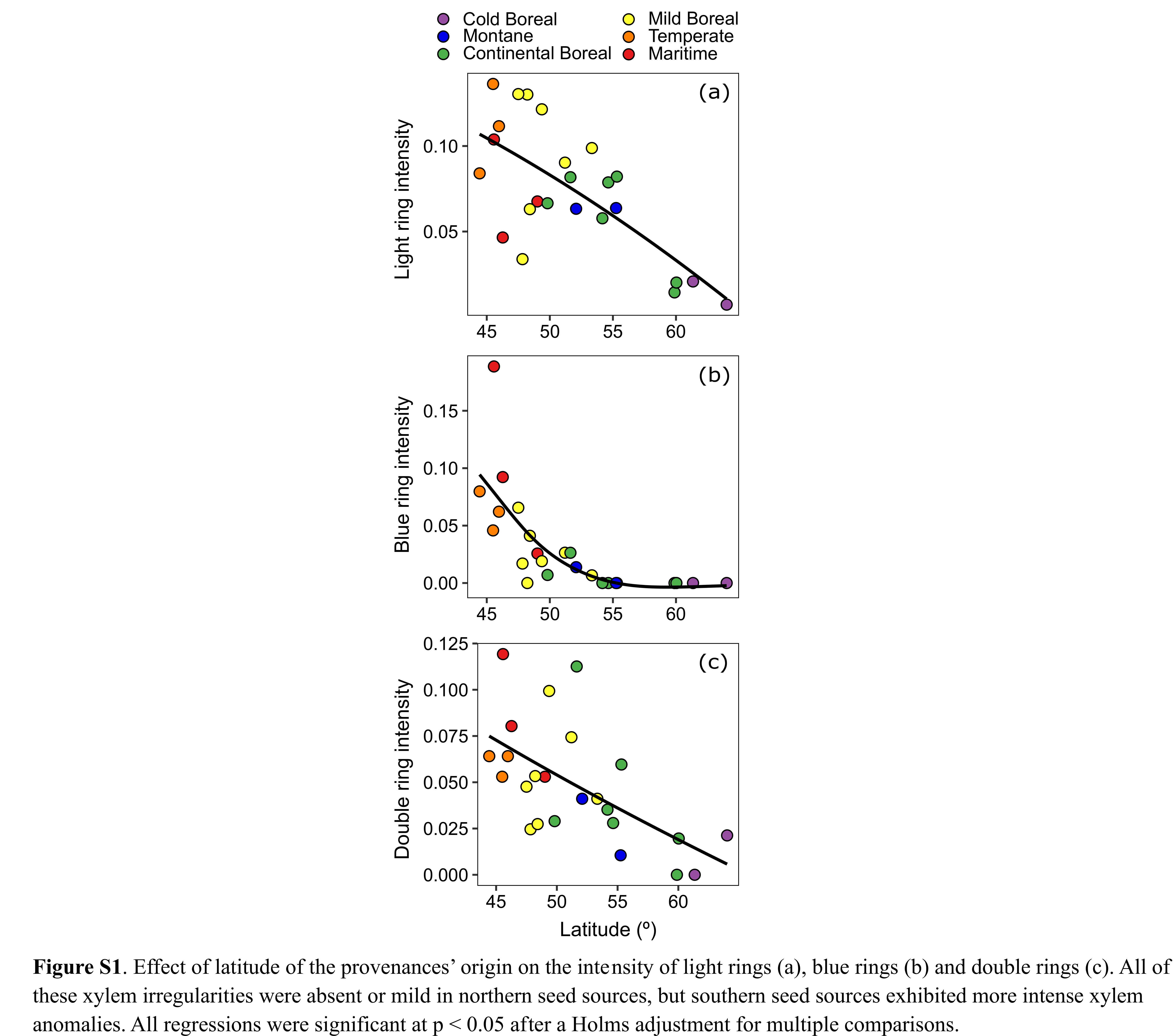

Supplement: Supplementary file 1 [file Image_1.JPEG]
